# Symmetrically Substituted Zn and Al Phthalocyanines and Polymers for Photodynamic Therapy Application

**DOI:** 10.3389/fchem.2021.647331

**Published:** 2021-06-24

**Authors:** Keshavananda Prabhu C. P., Manjunatha Nemakal, Muthumuni Managa, Tebello Nyokong, Lokesh Koodlur Sannegowda

**Affiliations:** ^1^ Department of Studies in Chemistry, Vijayanagara Sri Krishnadevaraya University, Ballari, India; ^2^ Department of Chemistry, Institute for Nanotechnology Innovation, Rhodes University, Makhanda, South Africa

**Keywords:** phthalocyanine, photodynamic therapy, singlet oxygen, quantum yield, infrared spectra

## Abstract

N4 macrocyclic complexes of Al and Zn phthalocyanines with symmetrical imine and imidazole moiety at the periphery were synthesized. The synthesized ligands, complexes, and polymers were purified and characterized to study the structure of the molecule. These synthesized complexes were used for photodynamic therapy application as the diamagnetic Zn and Al have the ability to produce and stabilize singlet oxygen species. The synthesized N4 molecules of aluminum iminomethoxy phenyl phthalocyanine and aluminum ethyl phenyl benzimidazolephthalocyanine showed better activity against MCF-7 cells. These results suggest that this assay may be used as an early biomarker of clinical response.

## Introduction

N4 macrocycles like phthalocyanines are conjugated, planar heterocyclic ligands, with an 18π electron system. A metal-N4 (M-N4) macrocycle complex provides tetradentate coordination properties to hold a metal in its central cavity (Sizun et al., 2012). Different metallophthalocyanines (MPcs) can be synthesized by altering the central metal atom at the core and/or substituting the different functional groups at the peripheral benzene rings (Nyokong and Gledhill, 2013). This will tune the properties of N4 macrocycles and also increase their potential applications in wide areas. These N4 macrocycles are basically known as azacolorants and find major applications as dyes and pigments. Nowadays, they are being used for diverse applications like bioactive transducers, corrosion inhibition, sensing, catalysis, fuel cells, photoconducting, and semiconducting (Malenka et al., 2009). M-N4 complexes are regarded as new generation photosensitizer materials/agents (Moreira et al., 2008). The photophysical and photochemical properties of M-N4 complexes strongly depend on the central metal atom/ion and conjugated aza-bridged tetra-pyrrolephthalocyanine ligand (Berridge et al., 2009). N4-macrocycles bearing bioactive and diamagnetic transition metals are being explored for the potential and most favorable properties in the biological application especially in bio-imaging, bio-sensing devices, and photodynamic therapy (PDT), in addition to staining and biological studies (Sibata et al., 2004). The symmetrically/unsymmetrically substituted derivatives of zinc (II) and aluminum (III) N4-complexes are emerging as promising candidates for PDT in cancer treatment (Imadadulla et al., 2019). PDT has many advantages over current cancer treatment modalities, such as surgery, chemotherapy, and radiation therapy (Moraes et al., 2010; Mugadza and Nyokong, 2010). Photosensitizer agents such as phenothiazine dyes, porphyrins, and phthalocyanine dyes have a macrocyclic structure with accessible tetrapyrrolic sites and the illumination of light radiation results in the triplet excited state generation (Zhang et al., 2008). The molecule gets excited to the triplet state when irradiated with light in the wavelength range 630–800 nm. In the triplet excited state, the photosensitizing drug has the ability to form highly reactive oxygen intermediate species known as singlet oxygen species. M-N4 macrocycles generate molecular oxygen intermediates like singlet oxygen or superoxide anion or hydroxyl radical on irradiation with light which are used in the photodynamic therapy (PDT) as a therapeutic effective stimulant (Bondavalli et al., 2009). According to the literature, MPcs have the capability to generate more reactive monovalent oxygen species by light interaction and this species exhibits a cytotoxic effect. The generated singlet oxygen is allowed to interact with tumor cells. Further, M-N4 compounds are known to be promising candidates for PDT because they can be efficiently incorporated into target cells, and they are nontoxic to healthy cells and exhibit a high yield of triplet states (Beheshtian et al., 2012; Bouvet et al., 2013; Keshavananda Prabhu et al., 2019a; Li et al., 2018; Veeresh et al., 2019).

**FIGURE 1:**
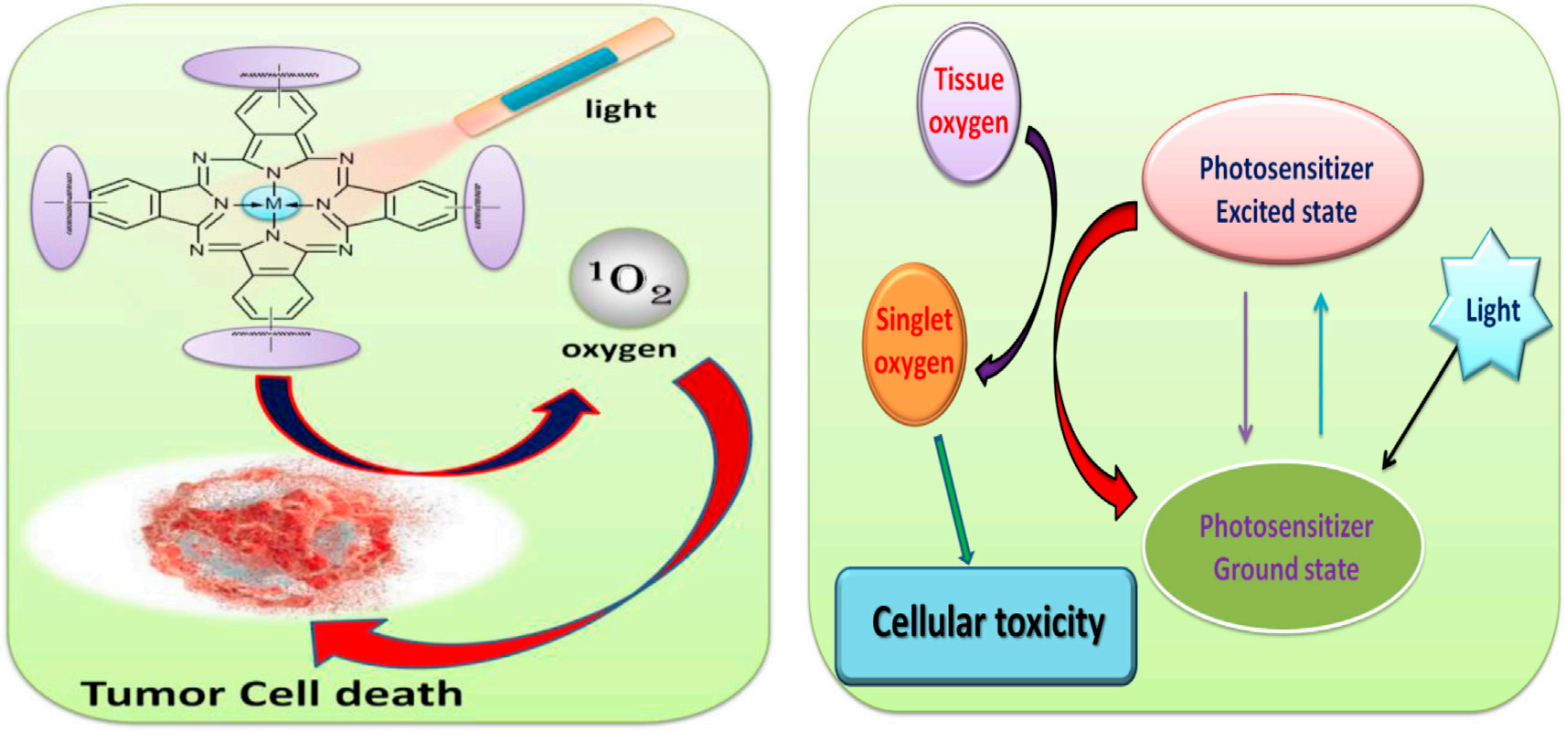
Schematic illustration for the application of phthalocyanine in photodynamic therapy study (PDT).

In this present work, the metallophthalocyanines of aluminum and zinc with different substituents have been synthesized for PDT study. The application of phthalocyanine in photodynamic therapy study (PDT) is showed in Figure 1. The diamagnetic metal-containing phthalocyanines are known to stabilize the excited triplet state as well as increase the quantum efficiency. Furthermore, molecules like imine and benzimidazole groups have shown various biological activities and are known to be biocompatible in nature. Further, the polymers are known to be nontoxic and act as a good scaffold for drug delivery and other applications. But there are very few reports of using polymers in PDT applications (Nyokong, 2003; Dube et al., 2018 and; Imadadulla et al., 2018). The conjugated ladder polymer has advantages over single-stranded conjugated polymer in terms of chemical resistance, thermal stability, mechanical strength, and greater molecular and structural order.

The present work mainly investigates and evaluates the photodynamic activity of the synthesized target strategic compounds on human breast adenocarcinoma Michigan cancer-7 cell line (MCF-7).

## Experimental details

### Materials

The chemicals used for the synthesis of phthalocyanine compounds and PDT studies were both obtained from Sigma-Aldrich or Merck and used as such without any purification.

Human breast adenocarcinoma Michigan cancer-7 cell lines (MCF-7) were purchased from Cellonex^®^. The Dulbecco’s Phosphate Buffered Saline and Dulbecco’s Modified Eagle’s Medium (DMEM) with and without phenol red were purchased from Lonza^®^. The heat-inactivated fatal bovine serum [FBS, 10% (v/v)] and 100 μg/ml–penicillin:100 unit/ml–streptomycin–amphotericin B mixture (PSA) as well as the neutral red cell proliferation reagent (WST) were all purchased from Biowest^®^.

### Synthesis of Precursor Compound

#### Synthesis of 2-{(E)-[(4-bromophenyl)imino]methyl} phenol **(IIIa)**

Compound **IIIa** was prepared by using a modified procedure reported in the literature (Keshavananda Prabhu et al., 2019b). The compound **I**, salicylic acid (2.25 g, 0.018 mmol), and compound **IIa**, p-bromoaniline (5 g, 0.0290 mmol), were mixed and taken in a round-bottom (RB) flask containing 25 ml methanol and refluxed at 60–70°C for 6 h in the presence of acetic acid catalyst. Then the crude compound was cooled and crushed ice pieces were added to precipitate the product and then filtered. The crude product was washed thoroughly with ice cold water and dried. Then the recrystallization of the compound **IIIa** was done using methanol.

Yield: 87%. Melting point: 108°C. Mol. wt.: 276.128. Anal. for the compound **IIIa** C_13_H_10_BrNO, Calc. (%): C, 56.52; H, 3.62; N, 5.07; O, 5.79; Br, 28.98. Found (%): C, 56.17; H, 3.19; N, 5.01. FTIR absorption bands [KBr (pellet), cm^−1^]: 730, 760, 841, 910, 970, 1242, 1340, 1560, 1600, 1750, 1903, 2218, 2508, 3100, and 3400. Mass (m/z): 276 (M^.^); 277 (M+1), and 278 (M+2).

#### Synthesis of 2-{(E)-[(4-methylphenyl)imino]methyl}phenol **(IIIb)**

The compound **IIIb** was synthesized by following the procedure for the preparation of **IIIa** and using compound **IIb**, p-toluidine, instead of compound **IIa** during the preparation.

Yield: 91%. Melting point: 115°C. Mol. wt.: 211.25. Anal. for the compound **IIIb** C_14_H_13_NO, Calc. (%): C, 79.60; H, 6.16; N, 6.63; O, 7.58. Found (%): C,79.93; H, 5.95; N, 6.18. FTIR absorption bands [KBr (pellet), cm^−1^]: 556, 700, 730, 901, 981, 1132, 1152, 1139, 1220, 1370, 1542, 1675, 1947, 2559, 3105, and 3412. Mass (m/z): 211 (M^.^)and 212 (M+1).

#### Synthesis of 2-(4-nitrophenyl)-1H-benzimidazole **(VIa)**

Precursor **VIa** was prepared by using a mixture of o-phenylenediamine (OPDA) (**IV**) (3.23 g, 0.0290 mmol), compound **Va**, and p-nitro benzoic acid (5 g, 0.0290 mmol) and taken in an RB flask containing 50 ml 4N HCl and 5 ml conc. H_2_SO_4_. The reaction mixture was refluxed at 80–90°C for 6 h and then the crude product was cooled. The crude product was thoroughly washed with water, dried, and the product was recrystallized using methanol.

Yield: 86%. Melting point: 108°C. Mol. wt. 239.23. Anal. for compound ***Via*** C_13_H_9_N_3_O_2_, Calc. (%): C, 65.27; H, 3.76; N, 17.57; and O, 13.38. Found (%): C, 64.97; H, 3.39; and N, 17.81. FTIR absorption bands [KBr (pellet), cm^−1^]: 729, 765, 840, 920, 968, 1230, 1340, 1515, 1670, 1896, 1913, 2228, 2558, 2905, 3025, and 3445. Mass (m/z): 239(M^.^) and 240 (M+1).

#### Synthesis of 2-[(E)-2-phenylethenyl]-1H-benzimidazole **(VIb)**

Compound **VIb** was prepared by using the same synthetic procedure as **VIa** and taking **Vb** instead of **Va** compound during the preparation.

Yield: 87%. Melting point: 102°C. Mol. wt.: 220.26. Anal. for the compound **VIb** C_15_H_12_N_2_, Calc. (%): C, 81.81; H, 5.45; and N, 12.72. Found (%): C, 81.36; H, 5.72; and N, 12.18. FTIR absorption bands [KBr (pellet), cm^−1^]: 556, 700, 848, 901, 980, 1130, 1165, 1190, 1219, 1377, 1415, 1542, 1675, 1947, 2559, 3000, 3006, and 3452. Mass (m/z): 220 (M^.^)and 221 (M+1).

#### Synthesis of 2,2'-(butane-1,4-diyl)bis(1H-benzimidazole) **(VIc)**

The target compound **VIc** was prepared by following the procedure for the synthesis of **VIa** and using **Vc**, that is, adipic acid compound instead of **Va** in the synthetic process.

Yield: 89%. Melting point: 105°C. Mol. wt.: 290.36. Anal. for the compound **VIc** C_18_H_18_N_4_, Calc. (%): C, 74.48; H, 6.20; and N, 19.31. Found (%): C,74.63; H, 5.95; and N, 19.18. FTIR absorption bands [KBr (pellet), cm^−1^]: 550, 705, 848, 901, 981, 1132, 1122, 1169, 1219, 1277, 1405, 1532, 1615, 1847, 2569, 3010, 3006, and 3400. Mass (m/z): 290 (M^.^); 288 (M-2).

#### Synthesis of 4-(2-{(E)-[(4-bromophenyl)imino]methyl}phenoxy)benzene-1,2-dicarbonitrile ligand **(VIIIa)**

Compound **VIIIa** was prepared by mixing compounds **IIIa** (2.0 g, 0.0094 mmol), 4-nitrophthalonitrile **(VII)** (1.63 g, 0.0094 mmol), and potassium carbonate (1.55 g, 0.011 mmol) (1:1:1.5) in an RB flask with dry dimethylformamide (DMF) (10 ml). The grounded mixture was stirred at room temperature for one day in the N_2_ atmosphere. The progress and completion of the reaction was monitored by thin layer chromatography (TLC). After the reaction completion, the crude product was cooled and poured into a beaker containing ice water to obtain the precipitate. The precipitate was filtered and washed with copious amount of deionized water. The compound **VIIIa** obtained was recrystallized from ethanol solvent and dried in vacuum.

Yield: 87%.Melting point: 260°C. Mol. wt.: 402.24. Anal. for compound **VIIIa** C_22_H_15_BrN_3_O, Calc. (%): C, 62.68; H, 2.98; N, 10.44; O, 3.98; and Br, 19.90. Found (%): C, 62.18; H, 2.52; and N,10.02. FTIR absorption bands [KBr (pellet), cm^−1^]: 759, 814, 937, 1006, 1100, 1295, 1356, 1380, 1471, 1541, 1610, 1693, 1926, 2242, 2910, 3100, and 3250. Mass (m/z): 402 (M^.^); 404 (M+2); and 405 (M+3).

#### Synthesis of 4-(2-{(E)-[(4-methylphenyl)imino]methyl}phenoxy)benzene-1,2-dicarbonitrile ligand **(VIIIb)**

Compound **VIIIb** was prepared by the procedure as described in the preparation of compound **VIIIa** and using compound **IIIb** instead of compound **IIIa.**

Yield: 90%. Melting point: 214°C. Mol. wt.:337.37. Anal. for compound **VIIIb** C_22_H_15_N_3_O: Calc. (%): C, 78.33; H, 4.45; N, 12.46; O, 4.74. Found (%): C, 77.96; H, 3.97; N, 12.12. FTIR absorption bands [KBr (pellet), cm^−1^]: 780, 828, 937, 951, 1091, 1114, 1251, 1306, 1570, 1541, 1665, 1782, 2235, 2894, 3066, and 3253. Mass (m/z): 337 (M^.^); 336 (M-1); and 335 (M-2).

#### *Synthesis of* 4-[2-(4-nitrophenyl)-1*H*-benzimidazol-1-yl]benzene-1,2-dicarbonitrile ligand**(VIIIc)**

Compound **VIIIc** was prepared by using the synthetic protocol explained for **VIIIa** and using **VIa** instead of **IIIa.** The crude compound was recrystallized from ethanol solvent system.

Yield: 92%. Melting point: 160°C. Mol. wt.: 367.36. Anal. for compound **VIIIc** C_21_H_13_N_5_O_2_, Calc. (%): C, 68.66; H, 3.54; N, 19.07; and O, 8.71. Found (%): C, 68.98; H, 3.82; and N,18.92. FTIR absorption bands [KBr (pellet), cm^−1^]: 759, 804, 930, 1016, 1120, 1290, 1355, 1375, 1471, 1540, 1665, 1683, 1926, 2240, 2835, 3066, and 3337. Mass (m/z): 367 (M^.^) and 368 (M+1).

#### Synthesis of 4-{2-[(E)-2-phenylethenyl]-1H-benzimidazol-1-yl}benzene-1,2-dicarbonitrile ligand **(VIIId)**

Similarly, the compound **VIIId** was obtained by using the above procedure of **VIIIa** and using **VIb** instead of **IIIa** compound during the preparation.

Yield: 89%. Melting point: 184°C. Mol. wt.: 346.38. Anal. for compound **VIIId** C_23_H_14_N_4_, Calc. (%): C, 79.76; H, 4.04; and N, 16.18. Found (%): C, 79.26; H, 3.97; and N, 15.82. FTIR absorption bands [KBr (pellet), cm^−1^]: 780, 828, 937, 951, 1091, 1104, 1256, 1326, 1580, 1541, 1665, 1712, 1900, 2232, 2894, 3056, and 3423. Mass (m/z): 346 (M^.^)and 348 (M+2).

#### Synthesis of [2,2']-(Butyl)bis[4-(benzimidazole-1-yl)-phthalonitrile] ligand **(VIIIe)**

The ligand **VIIIe** was obtained by following the above procedure of **VIIIa** and using **VIc** precursor instead of **IIIa** in the synthesis process.

Yield: 90%. Melting point: 214°C. Mol. wt.: 542.59. Anal. for compound **VIIIe** C_34_H_22_N_8_, Calc. (%): C, 75.27; H, 4.05; and N, 20.66. Found (%): C, 75.66; H, 3.97; and N, 20.92. FTIR absorption bands [KBr (pellet), cm^−1^]: 760, 821, 917, 955, 1084, 1124, 1266, 1316, 1585, 1542, 1685, 1782, 1899, 2245, 2890, 3020, and 3430. Mass (m/z): 542 (M^.^)and 543 (M+1).

### Synthesis of Substituted Aluminum and Zinc Phthalocyanine Complexes

#### Synthesis of zinc (II) tetra[4-(2-{(E)-[(4-bromophenyl)imino]methyl}phenoxy)] phthalocyanine (ZnTBrImPc) **(IXa)**

A grounded mixture of compound **VIIIa** (1.0 g, 0.0024 mmol), zinc chloride (0.08 g, 0.00062 mmol), dry 1-pentanol (25 ml), and catalytic amount of 1,8-diazabicyclo[5.4.0]undec-7-ene (DBU) were taken in an RB flask and refluxed with continuous stirring at 140°C for 1 day. Then, the dark blue–colored reaction mixture was cooled and filtered off and washed successively with cold and hot water. The blue-colored solid product was washed using hot ethanol, acetone, and then with hexane. The product was dried in the vacuum oven for 1 h at 50°C to obtain a bluish solid.

Yield: 85 %. Mol. wt.: 1703.40. Anal. for ZnTBrImPc **(IXa)** C_84_H_49_Br_4_N_12_O_5_Zn, Calc. (%): C, 59.89; H, 2.87; Br, 18.79; N, 9.86; O, 4.69; and Zn, 3.81. Found (%): C, 59.32; H, 2.56; N, 9.48; and Zn, 3.26. UV–VIS [DMSO, λ_max_, nm (log ɛ)]: 310 (4.49), 340 (4.53), 620 (4.79), 665 (4.82). FTIR absorption bands [KBr (pellet), cm^−1^]: 520, 670, 807, 910, 942, 1000, 1100, 1105, 1152, 1250, 1450, 1588, 1650, 2203, 2910, 3200, and 3395. Mass (m/z):1703 (M).

#### Synthesis of aluminum (III) tetra[4-(2-{(E)-[(4-methylphenyl)imino]methyl}phenoxy)] phthalocyanine **(IXb)**

The phthalocyanine complex **IXb** was prepared by adopting the procedure used for the preparation of **IXa** and using **VIIIb** and AlCl_3_ compounds instead of **VIIIa** and ZnCl_2_, respectively, during the synthetic protocol.

Yield: 85%. Mol. wt.: 1405.40. Anal. for AlTImPc**(IXb)** C_89_H_61_AlN_12_O_5_, Calc. (%): C, 76.01; H, 4.34; N, 11.95; O, 5.69; and Al, 1.92. Found (%): C, 75.72; H, 4.06; N, 11.28; and Al, 1.66. UV–VIS [DMSO, λ_max_, nm (log ɛ)]: 330 (4.51), 350 (4.54), 600 (4.77), and 670 (4.82). FTIR absorption bands [KBr (pellet), cm^−1^]: 520, 680, 740, 840, 955, 1020, 1100, 1247, 1319, 1380, 1428, 1490, 1620, 2009, 2849, 3000, and 3350. Mass (m/z): 1405 (M^.^) and 1402 (M-3).

#### Synthesis of zinc (II) tetra [4-[2-(4-nitrophenyl)-7,7a-dihydro-1H-benzimidazol-1-yl]] phthalocyanine (ZnTBImPc) (**IXc)**

The benzimidazole phthalocyanine complex **IXc** was prepared using the synthesis procedure as like **IXa** and using **VIIIc** instead of **VIIIa**.

Yield: 80%. Mol. wt.: 1526.78. Anal. for ZnTBImPc (**IXc**) C_92_H_56_N_16_Zn, Calc. (%): C, 76.08; H, 3.85; N, 15.43; and Zn, 4.47. Found (%): C, 75.85; H, 3.56; N, 15.08; and Zn, 4.11. UV-VIS [DMSO, λ_max_, nm (log ɛ)]: 295 (4.46), 357 (4.55), 600 (4.77), and 685 (4.83). FTIR absorption bands [KBr (pellet), cm^−1^]: 550, 680, 750, 807, 910, 942, 1091, 1100, 1105, 1152, 1247, 1453, 1580, 1796, 1803, 2203, 2600, 2940, and 3390. Mass (m/z): 1526 (M^.^); 1527 (M+1); and 1529 (M+3).

#### Synthesis of aluminum (III) tetra [4-{2-[(E)-2-phenylethenyl]-1H-benzimidazol-1-yl}] phthalocyanine **(IXd)**

The macrocyclic complex **IXd** was prepared by using the above procedure of **IXa** and using **VIIId** and AlCl_3_ instead of **VIIIa** and **ZnCl**_**2**_.

Yield: 85%. Mol. wt.: 1307.36. Anal. for AlTBImPc **(IXd)** C_84_H_48_AlN_16_, Calc. (%): C, 77.06; H, 3.66; N, 17.12;and Al, 2.06. Found (%): C, 77.62; H, 3.26; N, 16.98; and Al, 1.82 UV–VIS [DMSO, λ_max_, nm (log ɛ)]: 320 (4.50), 345 (4.53), 604 (4.78), and 660 (4.81). FTIR absorption bands [KBr (pellet), cm^−1^]: 560, 652, 742,769, 828, 960, 1191, 1107, 1247, 1280, 1328, 1486, 1620, 2209, 2840, 2920, and 3236. Mass (m/z): 1307 (M^.^)and 1305 (M-2).

#### Synthesis of zinc (II) tetra [4[2,2']-(butyl)bis[4-(benzimidazole-1-yl)]] polymer phthalocyanine**(IXe)**

The phthalocyanine polymer **IXe** was prepared by following the procedure of **IXa** and using **VIIIe** instead of **VIIIa**. The crude product obtained was a thick solution and the purified product was amorphous in nature.

Yield: 92%. Mol. wt.: [2283.90]_n_. Anal. for compound poly**(**ZnTBImPc) **(IXe)** [C_139_H_100_N_32_Zn]_n_, Calc. (%): C, 73.02; H, 4.37; N, 19.61;and Zn, 2.84. Found (%): C, 72.85; H, 4.96; N, 19.08; and Al, 2.62. UV-VIS [DMSO, λ_max_, nm (log ɛ)]: 325 (4.51), 349 (4.54), 610 (4.78), and 680 (4.83). IR absorption bands [KBr (pellet), cm^−1^]: 752,760, 841, 956, 1095, 1105, 1246, 1329, 1386, 1427, 1406, 1620, 2700, 2849, 2920, and 3441.

#### Synthesis of aluminum (III) tetra [4[2,2']-(butyl)bis[4-(benzimidazole-1-yl)]] polymer phthalocyanine **(IXf)**

Similarly, the compound **IXf** was prepared by using the procedure of **IXa** and reacting **VIIIe** and anhydrous AlCl_3_ metal salt instead of **VIIIa** and ZnCl_2_.

Yield: 89%. Mol. wt.: [2245.48]_n_. Anal. for compound poly**(**AlTBImPc) **(IXf)**, [C_139_H_100_N_32_Al]_n_: Calc. (%): C, 74.29; H, 4.45; N, 19.95; and Al, 1.20. Found (%): C, 73.82; H, 3.96; N, 19.58; and Al, 1.02. UV–VIS [DMSO, λ_max_, nm (log ɛ)]: 310 (4.49), 340 (4.53), 645 (4.80), and 690 (4.83). FTIR absorption bands [KBr (pellet), cm^−1^]: 740,789, 846, 970, 1051, 1120, 1247, 1369, 1480, 1499, 1620, 2029, 2840, 2917, and 3450.

### Characterization Methods

The elemental composition of the ligands and complexes were estimated by a Vario EL III CHNS elemental analyzer. A gravimetric procedure was employed to find the metal content in the macrocyclic complex. UV–Vis absorption spectra were performed using an FLMSO4808 Ocean optics spectrometer in the wavelength range 300–800 nm with 0.1 mM complex dissolved in DMSO. The KBr pellet technique was used to collect the FT-IR spectra from an PerkinElmer Spectrum-Two FT-IR spectrophotometer in the region 4000–500 cm^−1^. A GC/MS instrument of 6100 series from Agilent technologies was used for recording the mass data. An X-ray diffraction (XRD) profile was recorded by using a Bruker D8 Advance X-ray diffraction machine. Thermograms were recorded using an STA6000 machine to study the thermal stability in the temperature region 30–700°C (a heating rate of 10°C min^−1^).

The photodynamic therapy studies employed Modulight^®^ Medical Laser System (MLS) 7710–680 channel Turnkey laser system connected to a 2.3 W channel at 680 nm as a source of light for activation. The details of the designed source have been reported before (Dube et al., 2018; Imadadulla et al., 2019). The drug content in cell samples were determined by recording absorbance using the SpectraMax M3, Molecular Devices multi-well plate reader from Separations using the SoftMax Pro6.4 program.

### Procedure for *In Vitro* Dark Toxicity and Photodynamic Therapy Studies

In order to conduct the *in vitro* dark cytotoxicity experiments, the MCF-7 malignant neoplastic disease cells were civilized in Dulbecco’s changed Eagle’s medium (DMEM) containing 4.5 g/L glucose with L-glutamine (0.11 g/L) and phenol red, supplemented with 10% (v/v) heat-inactivated vertebrate calf blood serum (FCS) (50 ml), and a 100 unit/ml penicillin–100 μg/ml streptomycin–amphotericin B mixture. The cells were developed in 75-cm^2^ ventilated flasks (Porvair^®^), incubated at 37°C, and a five-hitter carbonic acid gas humidified atmosphere. Once 90–100% cell confluence was achieved, determined through microscopic examination, the cells were rinsed with Dulbecco’s changed phosphate buffered saline (DPBS). The cells were passed through routine trypsinization. Routine viability and cell enumeration were performed with the trypan blue dye exclusion assay (0.4% trypan blue solution) employing a hemocytometer. Cells were seeded at a cell density of 10,000 cells/well in supplemented DMEM-containing phenol red in 96-well tissue culture plates (Porvair^®^) and incubated in a very humidified atmosphere at 37°C and five-hitter carbonic acid gas for a day to foster cell attachment to the wells. The connected cells were rinsed with 100 μl DPBS once, followed by administration of 100 μl supplemented DMEM containing gradient concentrations of 0–100 μg/ml. Vehicle management was performed with a contemporary medium containing comparable amounts of dimethyl sulfoxide (DMSO) [1% (v/v)].

After 24-h incubation with supplemented DMEM with phenol red, cell proliferation neutral red chemical agent (WST-1) was used to find and quantify the extant cells. The WST-1 assay was employed to assess the toxicity and cell proliferation as per the manufacturer’s directions (Roche) using a Spectra Max M3, Molecular Devices multi-well plate reader from Separations employing the Soft Max Pro 6.4 program.

The percent cell viability was determined using Eq. 1 as follows:

$$\text{\% cell viability }=\;\frac{\text{Absorbance sample at }450\text{ nm}}{\text{Absorbance control at }450\text{ nm}}\times 100.$$ (1)

Here, the absorbance of sample is the cells containing **IXa-f** and control are the placebo cells containing 1% DMSO.

The PDT effects of various complexes were investigated by incubating cells as explained in the previous paragraph with varying concentrations of **(IXa-f)** and using the Modulight^®^ Medical optical device System (MLS) 7710–680 channel Turnkey laser optical device system. The irradiation time was 300 s to lead to irradiation doses of 100 J/cm^2^. After irradiation, the media were replaced with a recent one containing phenol red. The absorbance of the cells was measured with the SpectraMax M3, Molecular Devices multi-well plate reader from Separations using the SoftMax Pro6.4 program. There has been no change within the spectra of the Pcs following irradiation for PDT studies, therefore, confirming stability. The statistical analysis obtained from the three freelance triplicate experiments were analyzed with a three approach factorial multivariate analysis ANOVA to see the applied statistical variations between the *in vitro* cytotoxicity and photodynamic impact of the photosensitizers on MCF-7 cancer cells. Tukey’s HSD post hoc was accustomed to confirm the mean variations during the *in vitro* photodynamic impact of the photosensitizers on MCF-7 cancer cells. *p*-value of s < 0.05 was considered significant.

## Results and Discussion

**SCHEME 1:**
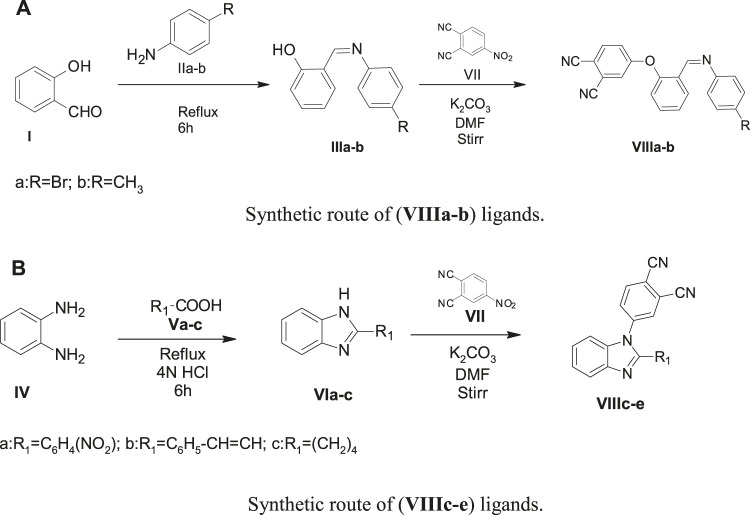
**(A)** Synthetic route of (**VIIIa–b**) ligands. **(B)** Synthetic route of (**VIIIc–e**) ligands.

The preparation of substituted phthalocyanines involved a sequential step-wise synthetic protocol. In Scheme 1A, amine-containing brominated or methyl compound was reacted with aldehyde-containing salicylaldehyde to yield the product bearing imine group, that is, Schiff’s base compounds **(IIIa-b)**. The benzimidazole precursor preparation is shown in Scheme 1B. The orthophenylenediamine (OPDA) (**IV)** was reacted with compounds having the –COOH group (**Va–c)** in the presence of 4N HCl to yield benzimidazole-containing precursors **(VIa-c)**.

**SCHEME 2:**
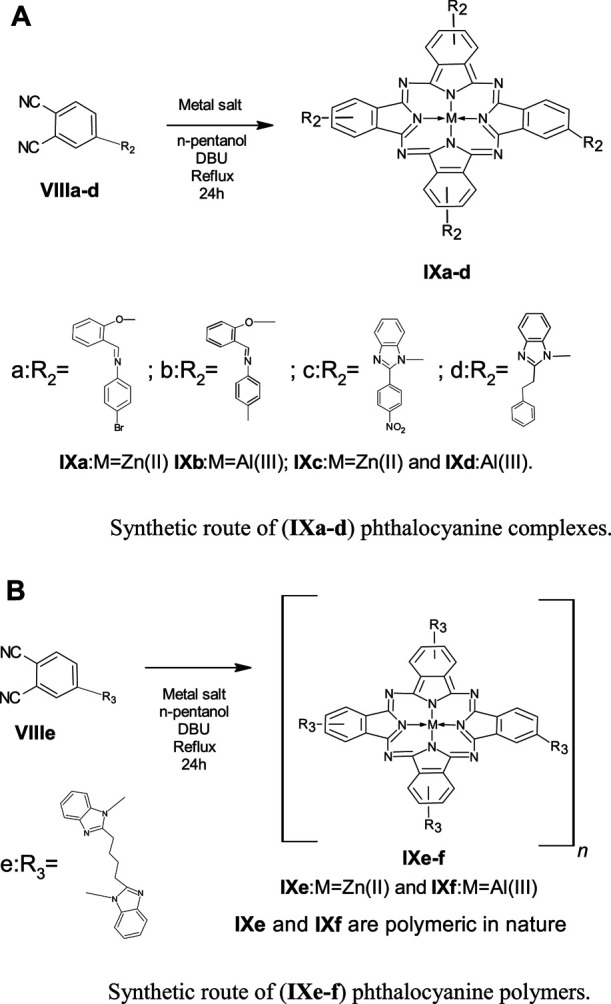
**(A)**Synthetic route of (**IXa–d**) phthalocyanine complexes. **(B)** Synthetic route of **(IXe-f)** phthalocyanine polymers.

The precursors **(IIIa-b)** and **(VIa-c)** undergo a nucleophilic displacement reaction with nitrophthalonitrile to form phthalonitrile ligands **(VIIIa-e).** In Scheme 2A and Scheme 2B, the ligands **(VIIIa-e)** undergo cyclization and condensation reactions with metal salt in the high-boiling liquid n-pentanol to form phthalocyanines **(IXa-f)**. The ring closure and cyclotetramerization of the phthalonitrile ligands take place in the presence of organic “super-base” 1,8-diazabicyclo(5.4.0)undec-7ene (DBU) yielding the macrocyclic complexes (**IXa-f)**. The ligands **(VIIIa-d)** have one phthalonitrile group in their structure and form monomeric phthalocyanines **(IXa-d)**, whereas **VIIIe** has two phthalonitrile moieties and on cyclotetramerization, it yields a polymeric phthalocyanine **(IXe-f)**.

The progress and completion of the reactions were monitored by TLC and the synthesized ligands were purified and characterized using melting point, elemental analysis, FT-IR, and mass spectroscopy. The phthalocyanine complexes were successfully purified to afford blue to green product with solubility in DMSO, DMF, and concentrated H_2_SO_4_.

### UV-Vis spectra

**FIGURE 2:**
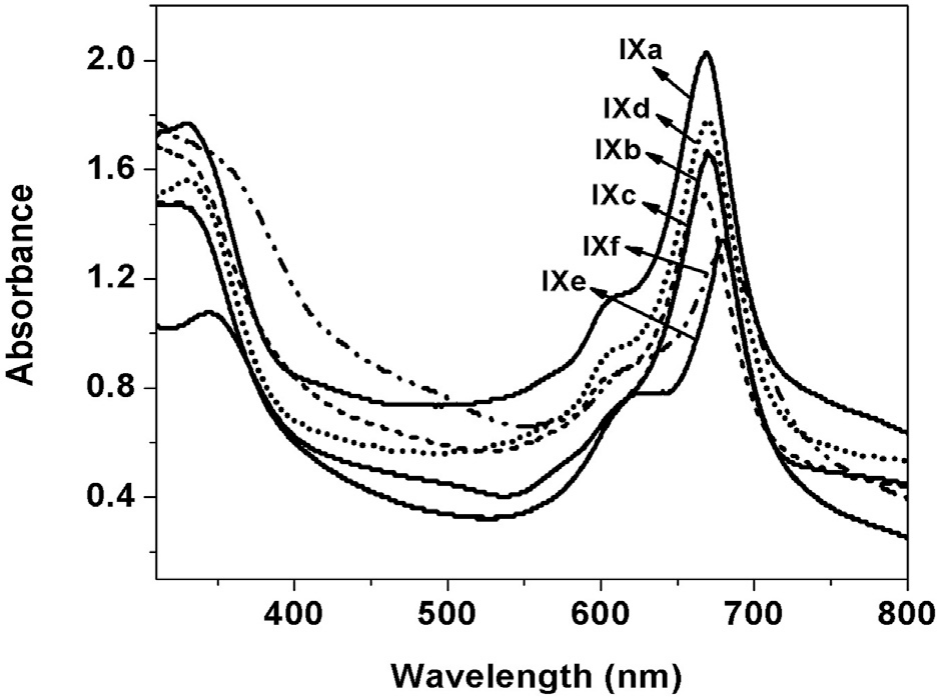
UV–visible spectra of (**IXa–f**) in DMSO.

The UV–visible spectra of all the monomeric Pcs were recorded by using 0.1 mM solution and polymeric Pc solution was prepared by dissolving 1 mg dry polymeric phthalocyanine in 5 ml DMSO. The UV–Vis spectra were recorded in the range 300–800 nm, Figure 2. All the synthesized Pcs showed absorption peaks at 320–350, 620–650 (shoulder peak), and 685–705 nm. The peak observed in the UV region at 320–350 nm is called as B-band and is attributed to n-π* transition. The intense Q-band was observed at 685–705 nm and the deep intense blue/green color of the phthalocyanine is due to this peak which arises because of the π–π* transition of the conjugated macrocycle (Keshavananda Prabhu et al., 2019a; Veeresh et al., 2019). The peak observed at 620–650 nm can be accounted for the oligomer and dimeric forms of the phthalocyanine macrocycle as well as vibronic fine structure of the Pc molecule. A bathochromic shift in the B- and Q-band was observed for the polymeric Pc, **IXe–f**, compared to monomeric Pc due to the increased conjugation in the polymeric structure. Further, polymeric Pcs showed broader and less intense peaks compared to monomeric Pcs.

### FT-IR Characterization

**FIGURE 3:**
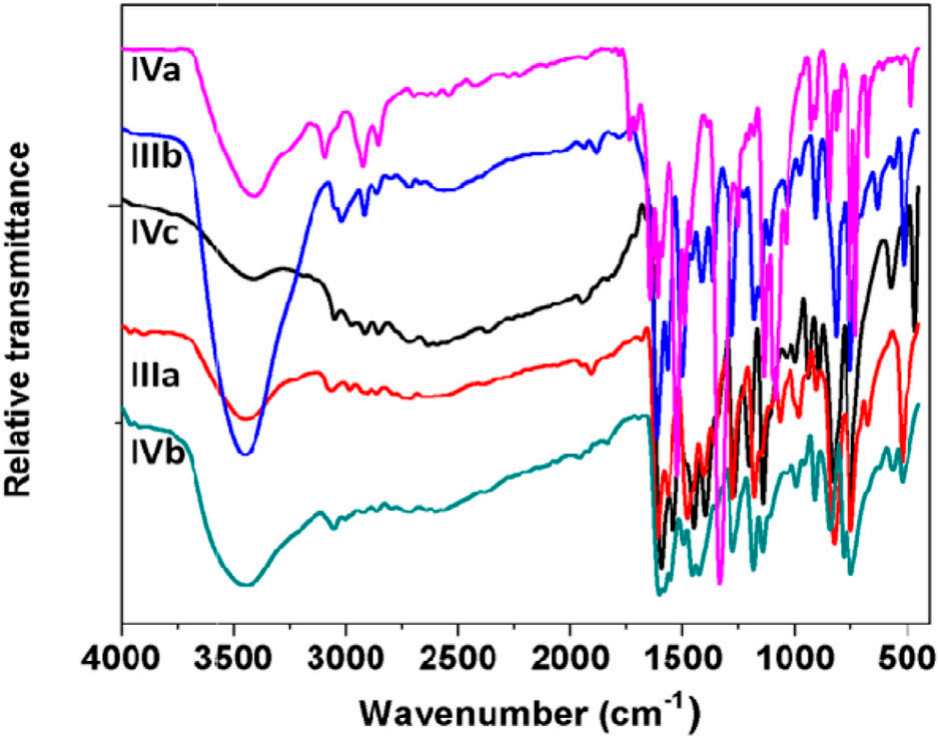
FT-IR spectra of **IIIa**, **IIIb**, and **VIa-c** precursors.

**FIGURE 4:**
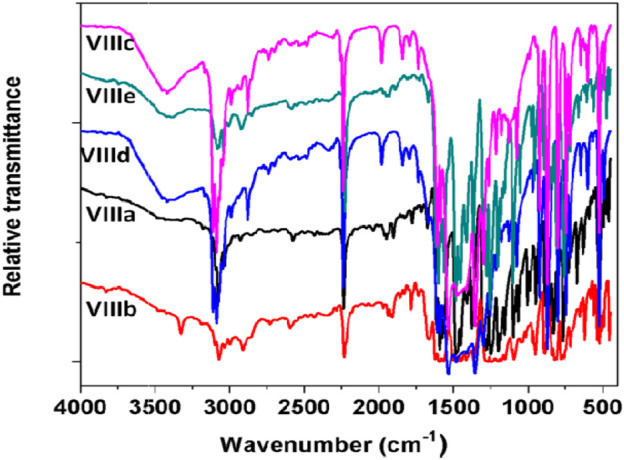
FT-IR spectra of (**VIIIa-e**) phthalonitrile ligands.

**FIGURE 5:**
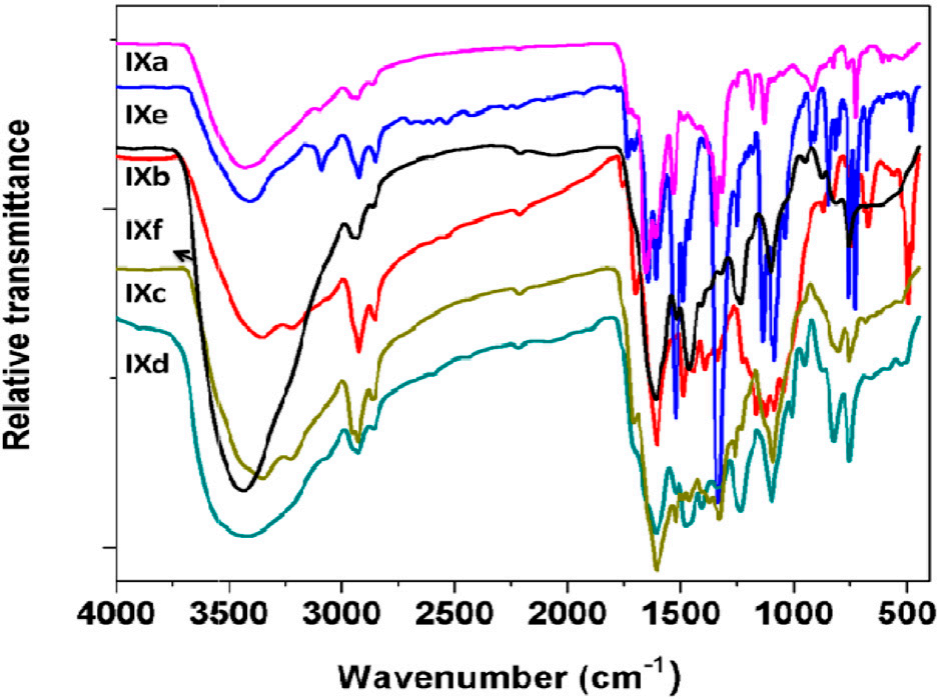
FT-IR spectra of (**IXa-f**) phthalocyanines.

FT-IR spectra for all the synthesized ligands and phthalocyanine complexes were recorded in the region from 500 to 4000 cm^−1^. The FT-IR spectra of precursor compounds **IIIa**, **IIIb**, and **VIa-c** are shown in Figure 3. All the compounds exhibited a vibrational band at 1540–1620 cm^−1^ which corresponds to C=C stretch and 2870–3020 cm^−1^ to C–H stretch. The precursors **IIIa** and **IIIb** showed broad peaks at 3100–3450 cm^−1^ which can be assigned to the –OH group. A weak peak was observed around 3200 cm^−1^ for **VIa-c**, which can be attributed to secondary amine. Figure 4 shows FTIR spectra of the ligands **VIIIa-e** showed a sharp peak around 2250 cm^−1^ corresponding to the –CN group. The appearance of –CN group and disappearance of the –OH or –NH peak in the ligands confirm the formation of the conjugated phthalonitrile ligands. In addition to TLC, IR was useful in the successful monitoring of the completion of the reaction to form cyano ligands from their respective hydroxy or imidazole precursors by carefully monitoring the evolution of the cyano group peak at ∼2250 cm^−1^ with the complete diminishment or disappearance of the hydroxy or –NH group at ∼3300 or 3200 cm^−1^. All the synthesized monomeric and polymeric phthalocyanine complexes displayed peaks at 745–760, 875–895, 965–990, 1004–1110, and 1111–1135 cm^−1^ which can be related to the phthalocyanine skeletal vibrations (Keshavananda Prabhu et al., 2019b; Manjunatha et al., 2019). Additionally, the IR spectra of the Pcs did not display the peak for the –CN group informing the utilization of cyano groups to form the monomeric and polymeric phthalocyanine system. Figure 5 shows FTIR spectra of the polymeric Pcs **IXe-f** and showed a very weak cyano peak at 2250 cm^−1^ which is due to the peripheral –CN peak of polymeric Pcs. The characteristic peaks of the polymeric Pcs were lesser in intensity as well as broader in nature than the monomeric Pcs.

### Mass Spectra

**FIGURE 6:**
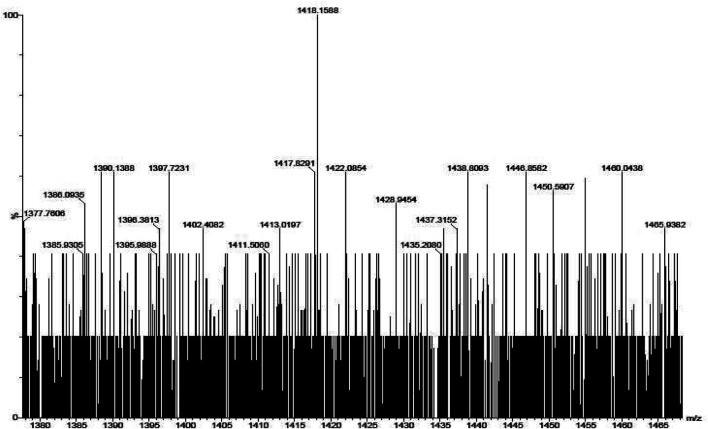
Mass spectrum for phthalocyanine **IXb** with mass fragment (M-3) = 1402.

**FIGURE 7:**
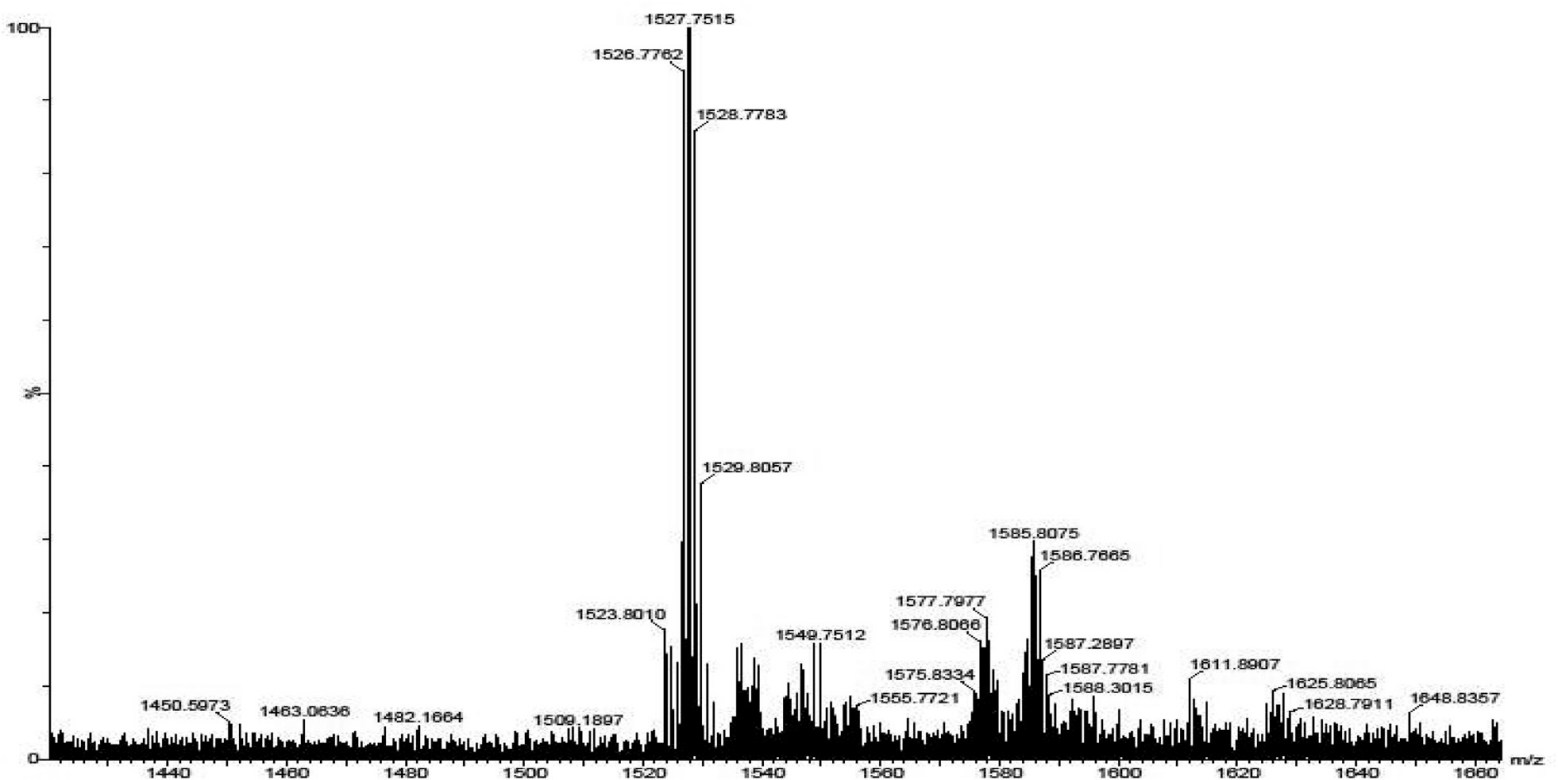
Mass spectrum for phthalocyanine **IXc** with mass fragment (M^.^) = 1526; (M+1) = 1527; and (M+3) = 1529.

**FIGURE 8:**
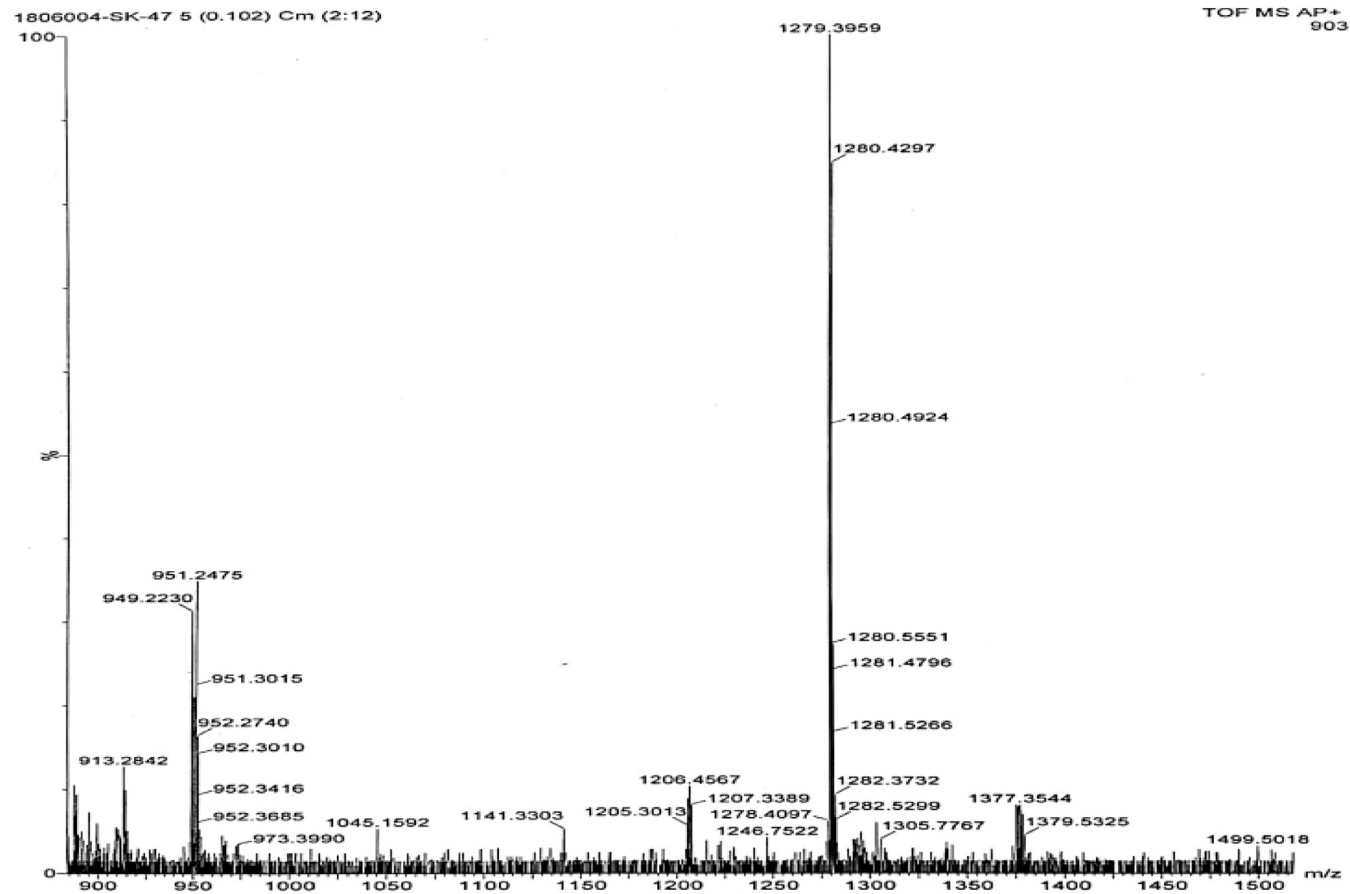
Mass spectrum for phthalocyanine **IXd** with mass fragment (M-2) = 1305.

The mass spectra of the precursor, ligand, and complexes were recorded to get information related to molecular weight and fragmentation pattern of these compounds. The mass spectra of precursors (**IIIa**, **IIIb**, **VIa**, **VIb**, and **VIc**), ligands (**VIIIa**, **VIIIb**, **VIIIc**, **VIIId**, and **VIIIe**) are presented in Supplementary Figure S1–S10; and monomeric complexes (**IXb**, **IXc**, and **IXd**) are presented in Figure 6, Figure 7, and Figure 8. The synthesized ligands showed (M, M^+^, or M^−^) molecular ion peaks in mass spectra which are well coinciding with the theoretical molecular weight of the compounds. Some ligands showed fragment ion peaks, which are responsible for the detachment of one part of the molecule from the ligand. Phthalocyanine molecules are one of the most stable structures known. The mass spectra of the synthesized monomeric phthalocyanine complexes are characterized by many competitive and consecutive pathways, thus forming many respective fragment ions. The molecular ion peaks observed for precursors, ligands, and complexes and their theoretical molecular weights are given in the respective synthetic section.

### X-Ray Diffraction Studies

The powder X-ray diffraction patterns for the synthesized complexes were recorded in 2θ range of 5–70^o^ as shown in Supplementary Figure S11. The patterns were found to be highly noisy, and the broader nature of the peaks revealed that the synthesized Pc compounds are highly amorphous in nature. Among all the synthesized Pcs, **IXb** was found to be partially crystalline in nature with few sharp peaks in the X-ray diffraction profile.

### Thermogravimetric Analysis (TGA) Studies

Thermogravimetric studies yield information related to the thermal stability and the decomposition characteristics of the synthesized complexes. The thermograms for the phthalocyanine complexes were recorded in the temperature range 30–700°C. The thermograms obtained for the thermal behavior of the different phthalocyanines are shown in Supplementary Figure S12. The nature of thermograms indicates that the Pc complexes degrade mainly in three steps in air atmosphere. In the first step, mass loss in the region below 175°C is due to the loss of volatile components, water, and moisture from the sample with an exothermic decomposition. In the second step, the terminal substituents get detached in the temperature region of 300–400°C. The final decomposition step occurs fast in oxidizing atmosphere and results in the accelerated loss of mass corresponding to the degradation of the phthalocyanine structure after 400°C. The stable and final product was formed after the decomposition of the complexes in the oxidizing atmosphere and the amount of the stable product is equivalent to the mass of metal oxide. Polymeric Pcs (**IXe-f**) showed better thermal stability than monomeric Pcs which may be accounted for the extended conjugation and delocalization of π electrons (Oni and Nyokong, 2001; Durmuş and Ahsen, 2010; Manjunatha et al., 2018; Keshavananda Prabhu et al., 2019b).

### Photodynamic Therapy Studies

The group of phenthiazine, pyrrol, and isoindole derivatives of macrocyclic compounds such as porphyrins and phthalocyanine dyes have the organic heterocyclic chelated structure. When these compounds are irradiated with visible light, these macrocyclic complexes acquire triplet excited states (compound has the maximum absorbance at 650–680 nm) and show photosensitizer behavior. This generates reactive oxygen species, efficiently affects the target cells, and is nontoxic to the healthy cells. This is the main phenomenon of photodynamic therapy (PDT). The PDT properties mainly depend on the structure of the Pc and the central metal atom. In the present study, we utilized the diamagnetic central metal atom, especially comparison of the Al(III) and Zn(II) phthalocyanines were made. According to the obtained data and literature data, both Al(III) and Zn(II) complexes exhibit the most favorable PDT properties. The phototoxic effect of Al(III) complexes are high efficacy and high susceptibility to *in vitro* photodynamic therapy and it showed an increased phototoxic effect compared to Zn(II) phthalocyanine complexes (similar concentration of both metal complexes used to study) that induce the release of activated oxygen species to inhibit the parasite.

**FIGURE 9:**
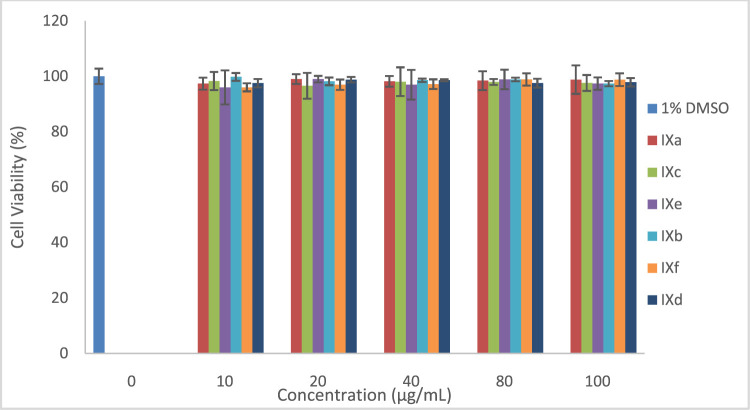
Dark toxicity plots for Pcs at 0, 10, 20, 40, 80, and 100 μg/ml.

Here, the properties of monomeric and polymeric phthalocyanines of Zn and Al have been studied and compared. Dark toxicity studies of the molecules were performed at 0, 10, 20, 40, 80, and 100 μg/ml, Figure 9. At all concentrations, the cell viability percent were all above 98%. *In vitro* dark toxicity is undesirable for photosensitizers aimed for use in PDT. The dark toxicity of the photosensitizers was determined in 1% DMSO which was used as the stock diluent. DMSO (1%) used had an negligible effect on the cells seen on this work and has also been reported (Managa et al., 2019; Nene et al., 2019).

**FIGURE 10:**
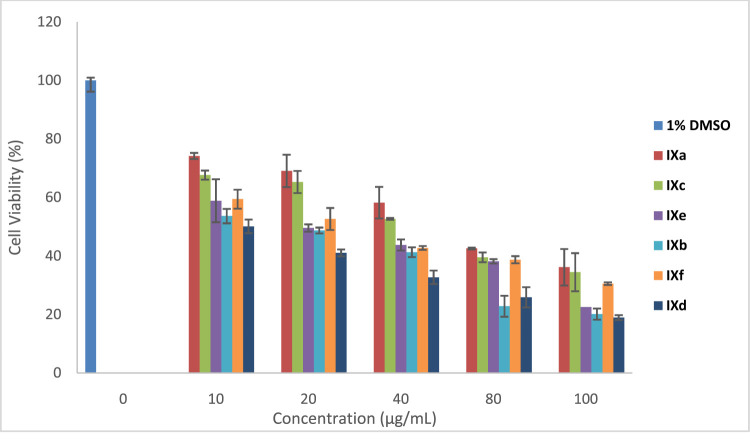
PDT for Pcs at 0, 10, 20, 40, 80, and 100 μg/ml.

The photodynamic therapy activities of the Pcs were evaluated on the MCF-7 cell lines *in vitro*. The cells were grown to 100% confluency, with approximately a 10^5^ cell unit which was counted using a hemocytometer. The Pcs were then inoculated at different concentrations ranging from 0 μg/ml–100 μg/ml. The cells were incubated under growth conditions over 24 h to allow for photosensitizer uptake. The activity of the molecules was tested and as seen in Figure 10, the activities of the molecules increased upon irradiation and increased with the increase in concentration. Molecules **IXb** and **IXd** showed the highest activity against MCF-7 cell lines *in vitro* as compared to other molecules.

The structural alteration at the phenylene ring and change in the metal atom in the cavity of the macrocyclic Pc ring results in altered properties and in turn changes in *in vitro* and biological activities. This leads to differential ability to generate reactive oxygen species (ROS) on irradiation (Çakır et al., 2015). ROS species in cellular biology is responsible for functions related to toxicity, cell death, proliferation, cell signaling, cellular pathophysiology, and metabolism. As the functionality differs, its reactivity also differs in *in vitro* studies (Sobotta et al., 2018). PDT primarily depend on the cell which is used for the examination, types of the Pcs, cell uptake, intensity of light, Pc’s incubation time, or localization in the target cell (Paquette et al., 1988; Rosenthal and Ben-Hur, 1995; Escobar et al., 2012). The above-mentioned features are possessed by Al(III) phthalocyanine complexes and hence improve the results of PDT activity. The amphiphilic nature of Al(III) Pc and its help in cellular uptake, Pc localization, influencing the higher excited triplet state yield, and lifetime, with no aggregation of the Al(III) complex, have a major influence on better PDT activity. Zn(II) complexes show hydrophobicity, thereby forming aggregation and reducing the lifetime of the triplet state; hence, the cytotoxic activity is less compared to the Al(III) macrocyclic complexes (Escobar et al., 2012).

Furthermore, cell survival depends on the dose concentration and on the nature of the substituent at the periphery of Pc ring (Hofman et al., 2007). Complexes **IXb** and **IXd** contain Al metal, but the difference in their *in vitro* activity can be explained based on electron-donating toluene and bulky groups present in **IXb** are able to prevent the aggregation and in turn enhance the absorption of radiation and ROS generation (Murphy et al., 1999; Huang et al., 2018). However, **IXd** has an electron withdrawing benzimidazole substituent which may not prevent the aggregation as like the electron-donating toluene group and hence lesser *in vitro* activity. The XRD spectrum of **IXb** is more crystalline than that of **IXd**, indicating less aggregation in **IXb** than that of **IXd**. Though **IXf** contains Al (**III**) metal ion, the molecule is polymeric in nature and in general, polymeric molecules are aggregated compared to monomeric molecules. Hence, **IXf** showed lesser *in vitro* activity than **IXb** and **IXd**.

Monomeric aluminum phthalocyanine treatment showed a higher phototoxic effect than Zn monomeric and Zn and Al polymeric phthalocyanines. This result could be attributed to the amphiphilic properties of AlPc that could facilitate cell uptake and intracellular localization, contrary to the hydrophobicity of ZnPc. The AlPc used in this study was soluble in culture medium, whereas ZnPc was less soluble. In general, phthalocyanines are prone to self-aggregation and dimers are reported to be inactive or much more inefficient than monomers as photosensitizers (Bonnett and Martínez, 2001). In water, ZnPc displays a strong tendency to form aggregates as a result of the propensity of the large hydrophobic skeleton to avoid contact with the aqueous medium. On the other hand, it is known that the central metal and ligand play a crucial role in the photobiological activity influencing the excited triplet state yield and lifetime (Rosenthal and Ben-Hur, 1995). Another important reason which could justify the difference in AlPc vs. ZnPc activity against parasites was the type of illumination system used in this study. It could be possible to obtain a higher ZnPc activity using a wide range of wavelength for illumination.

## Conclusion

Novel macrocyclic imine and imidazole derivatives of aluminum and zinc MN4 complexes were prepared with a good yield. These synthesized compounds were characterized using analytical techniques to confirm the purity and formation of the products. All the Pcs showed a significant PDT activity as compared to the *in vitro* dark cytotoxicity behavior. The activities of the molecules increased upon irradiation and also increased with an increase in concentration. Molecules **IXb** and **IXd** showed the highest activity against MCF-7 cell lines *in vitro* as compared to other Pc molecules.

## Data Availability

The original contributions presented in the study are included in the article/Supplementary Material, and further inquiries can be directed to the corresponding authors.
