# Supplementary material for: Symmetrically Substituted Zn and Al Phthalocyanines and Polymers for Photodynamic Therapy Application
Source: Front Chem. 2021 Jun 24;9:647331. doi: 10.3389/fchem.2021.647331 (PMC8263923; doi:10.3389/fchem.2021.647331)
Supplement: Supplementary file 1 [file DataSheet1.docx]

**Supplementary Information**

**Symmetrically substituted Zn and Al phthalocyanines and polymers for photodynamic therapy application**

Keshavananda Prabhu C P^†^, Manjunatha Nemakal^†^, Muthumuni Managa^‡^, Tebello Nyokong*^‡^_,_ Lokesh Koodlur Sannegowda*^†^,

^†^Department of Studies in Chemistry, Vijayanagara Sri Krishnadevaraya University, Vinayakanagara, Ballari-583105, Karnataka, India

^‡^Institute for Nanotechnology Innovation, Department of Chemistry, Rhodes University, Makhanda, 6140, South Africa

^*^Corresponding authors: [kslokesh@vskub.ac.in](mailto:kslokesh@vskub.ac.in);

[T.nyokong@ru.ac.za](mailto:T.nyokong@ru.ac.za)


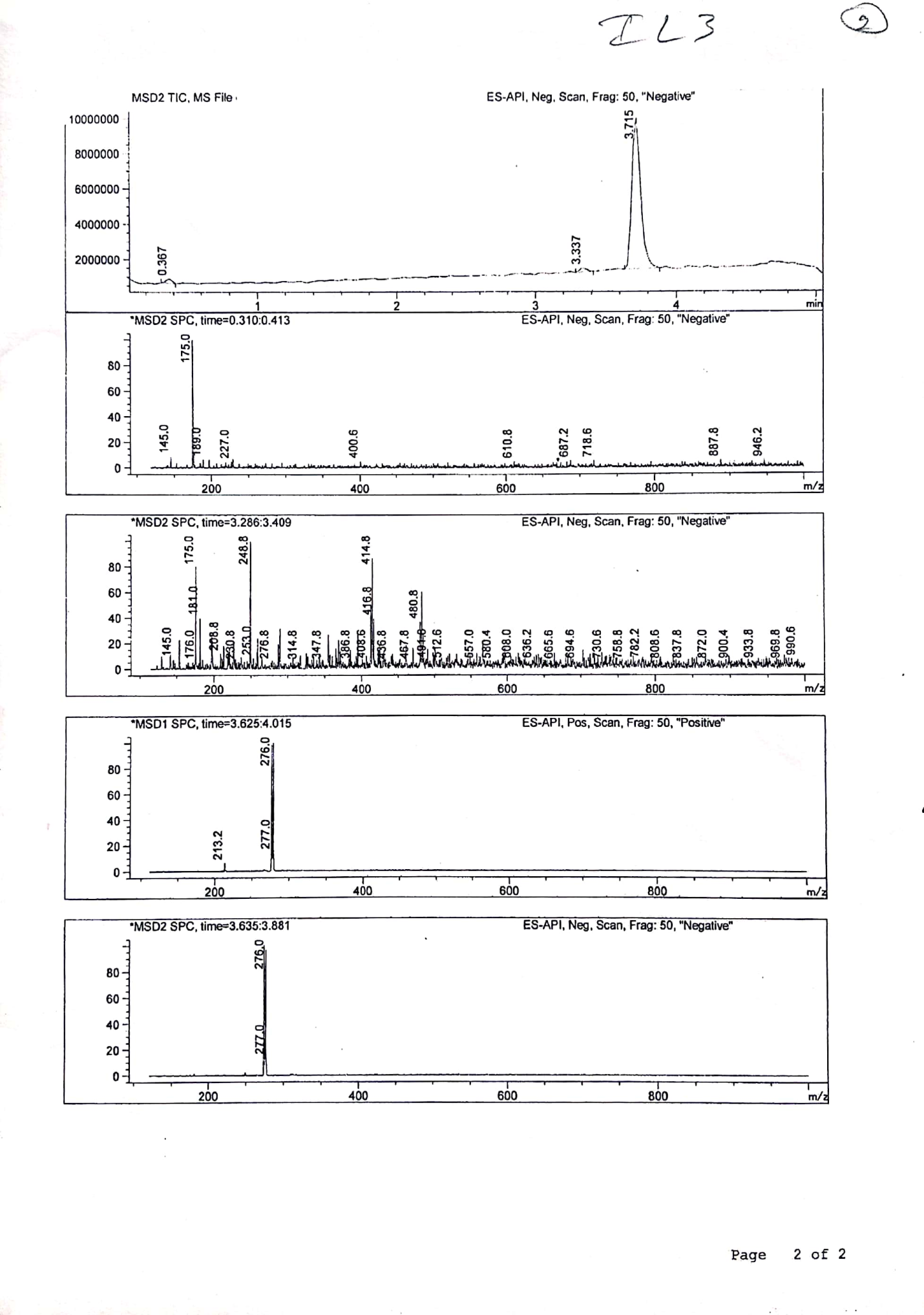


**Figure S1.** Mass spectrum of precursor **IIIa** with mass fragment M^.^ = 276 and (M+1) =277.


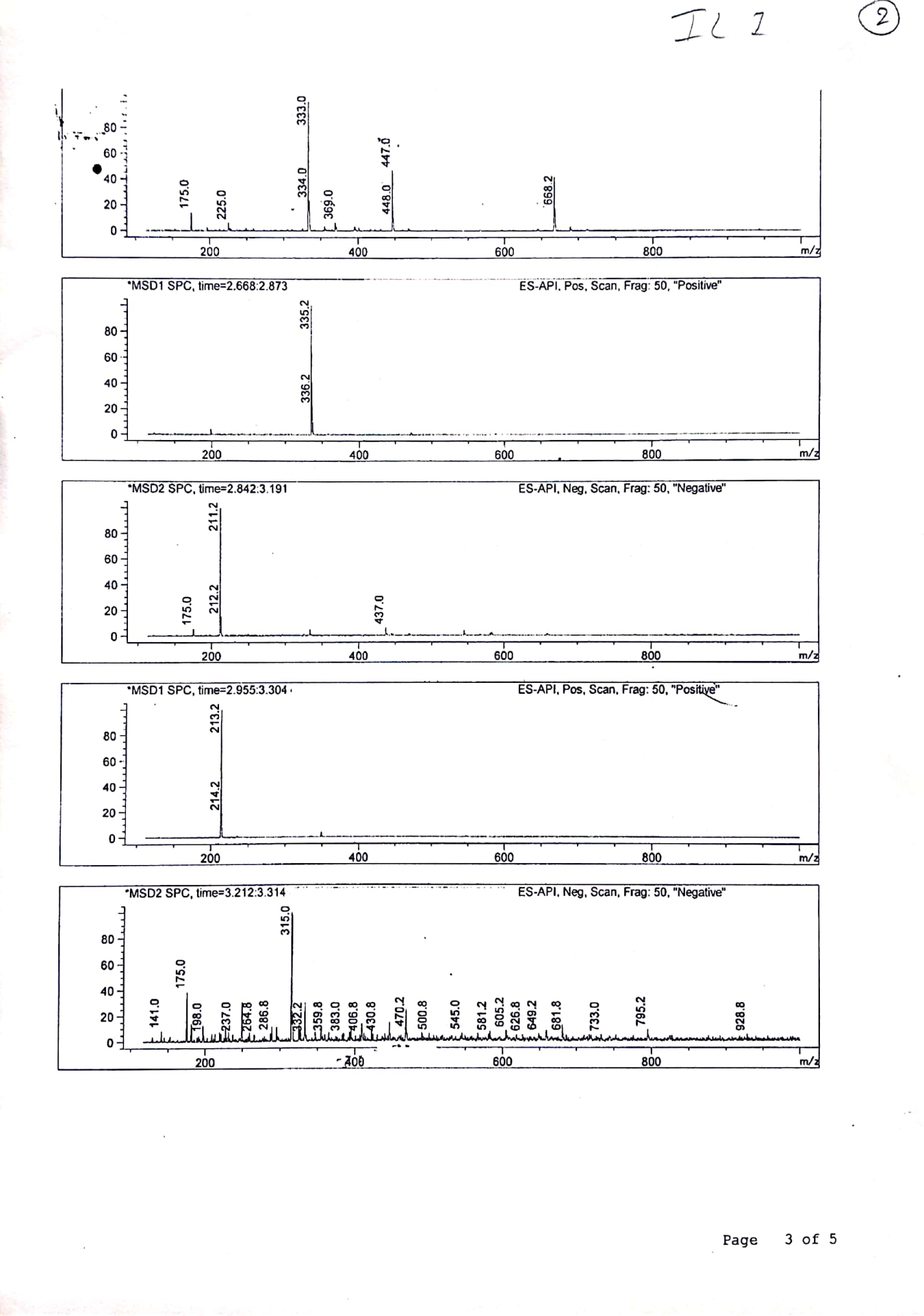


**Figure S2.** Mass spectrum of precursor **IIIb** with mass fragment M^.^ = 211 and (M+1) =212.

**Figure S3.** Mass spectrum of precursor **VIa** with mass fragment (M^.^)= 239; (M+1) =240.

**Figure S4.** Mass spectrum of precursor **VIb** with mass fragment M^.^ = 220 and (M+1) =221.


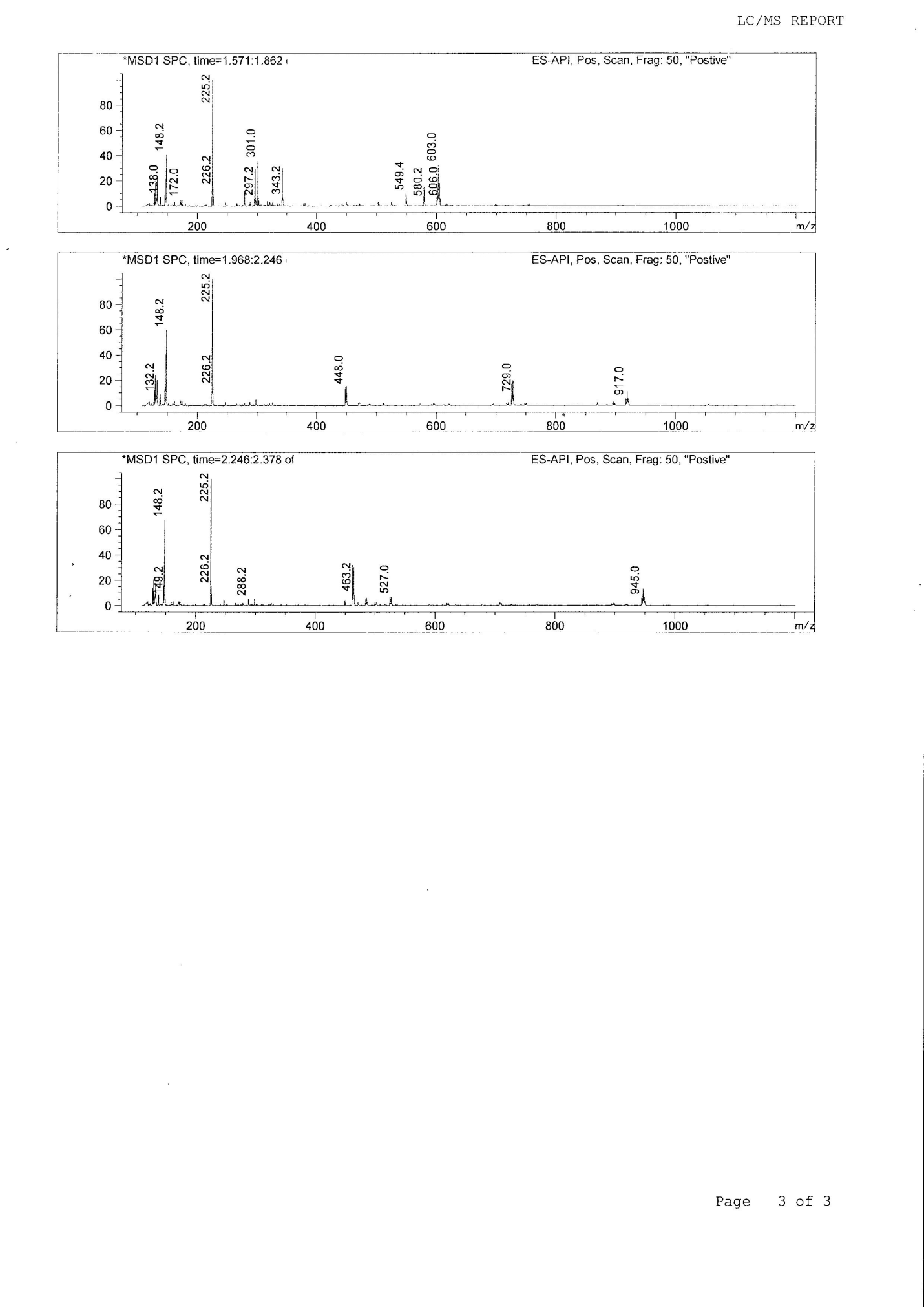


**Figure S5.** Mass spectrum of precursor **VIc** with mass fragment M^.^ = 290 and (M-2) =288.


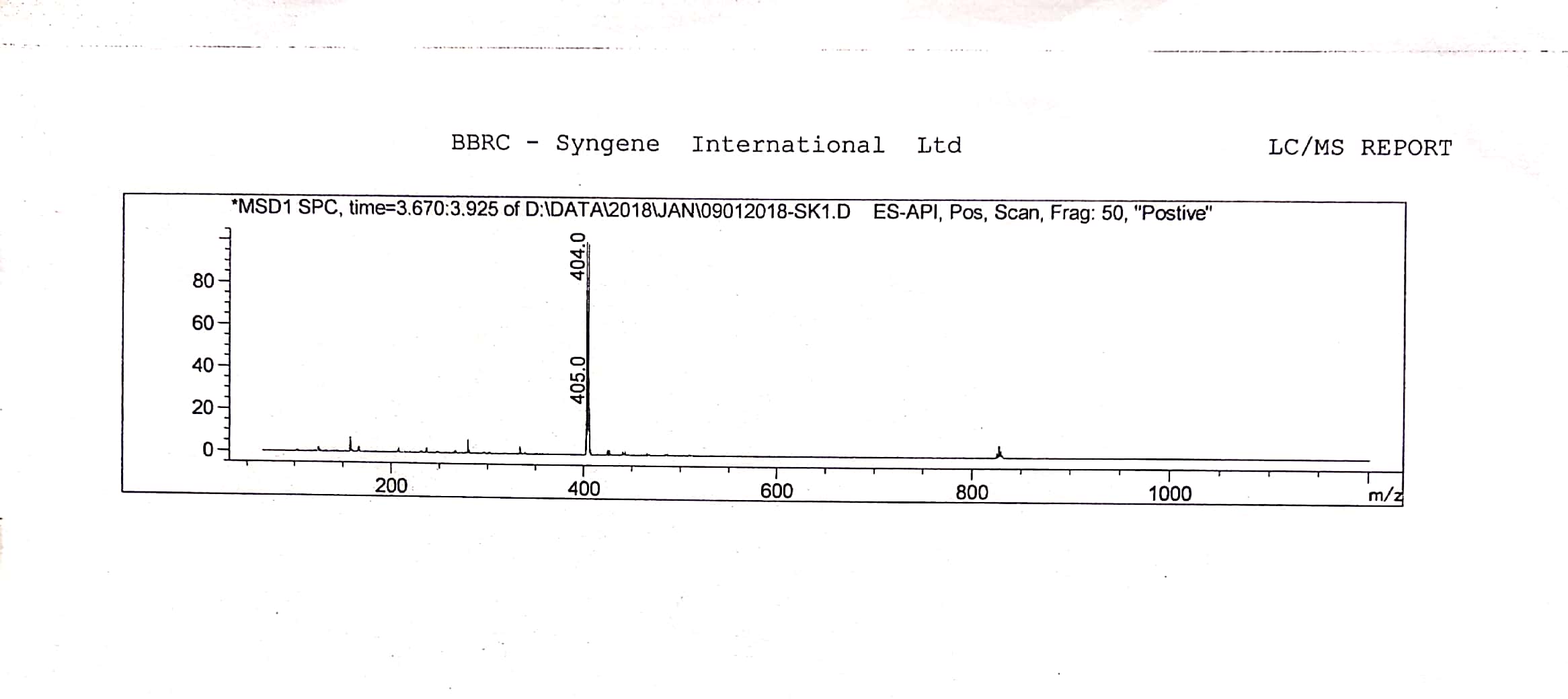


**Figure S6.** Mass spectrum of ligand **VIIIa** with mass fragment (M+2) =404 and (M+3) =405.


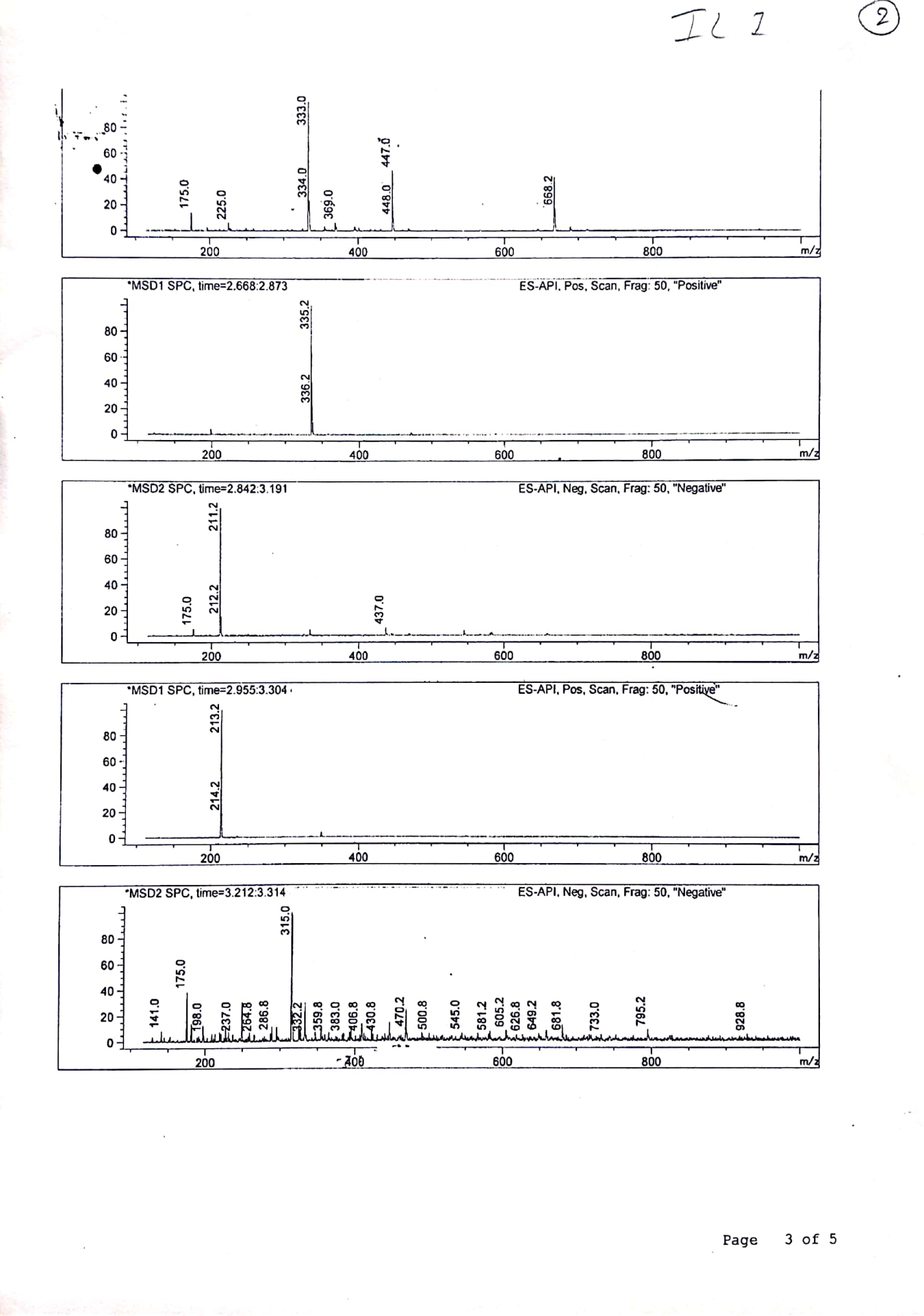


**Figure S7.** Mass spectrum of ligand **VIIIb** with mass fragment (M-1) =336; (M-2) =335.

**Figure S8.** Mass spectrum of ligand **VIIIc** with mass fragment M^.^ = 367; (M+1) =368.

 **Figure S9.** Mass spectrum of ligand **VIIId** with mass fragment M^.^ = 346; (M+2) =348.


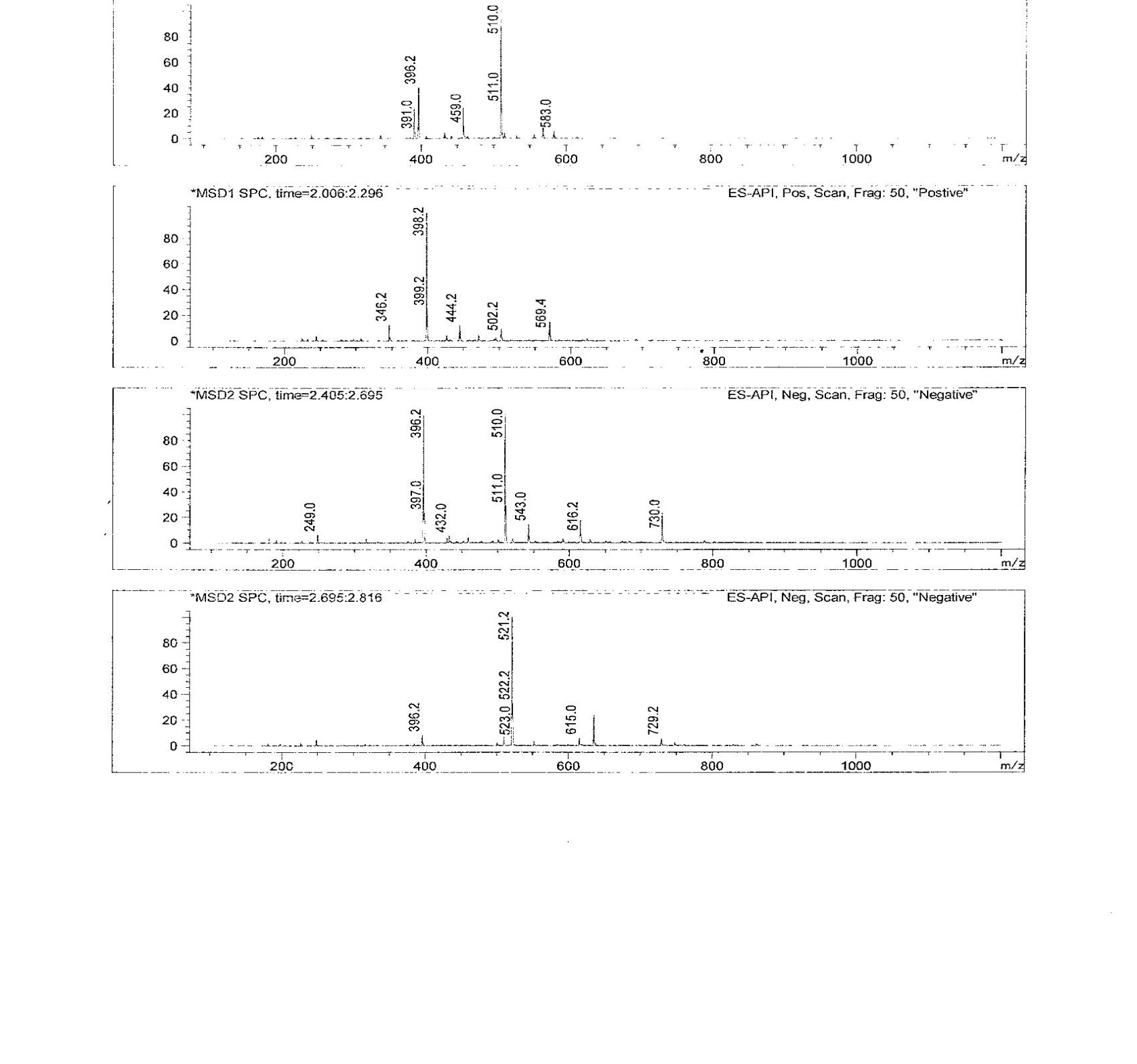


**Figure S10.** Mass spectrum of ligand **VIIIe** with mass fragment M^.^ = 542; (M+1) =543.

**
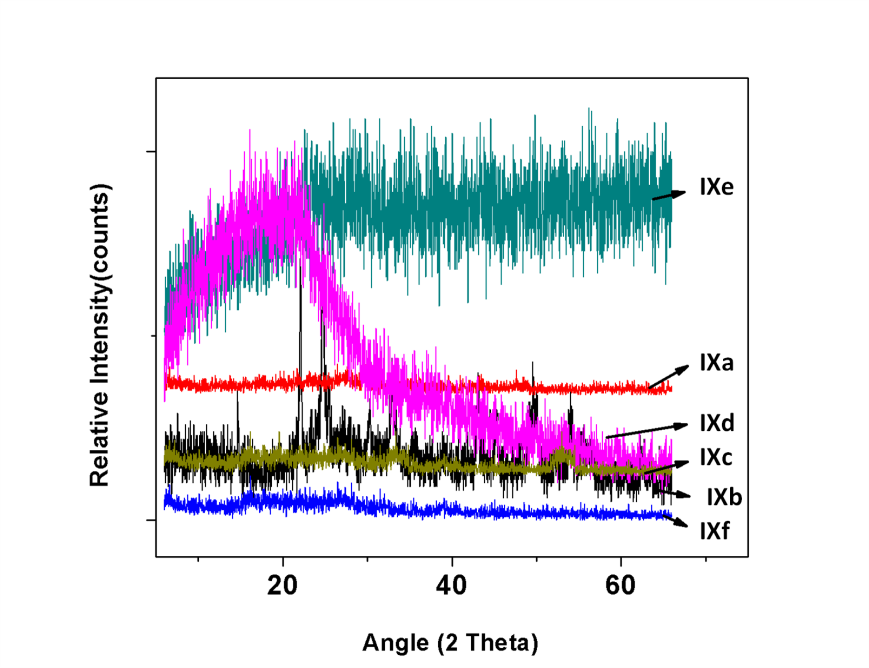
**

**Figure S11.** PXRD spectra of **(IXa-f).**

*
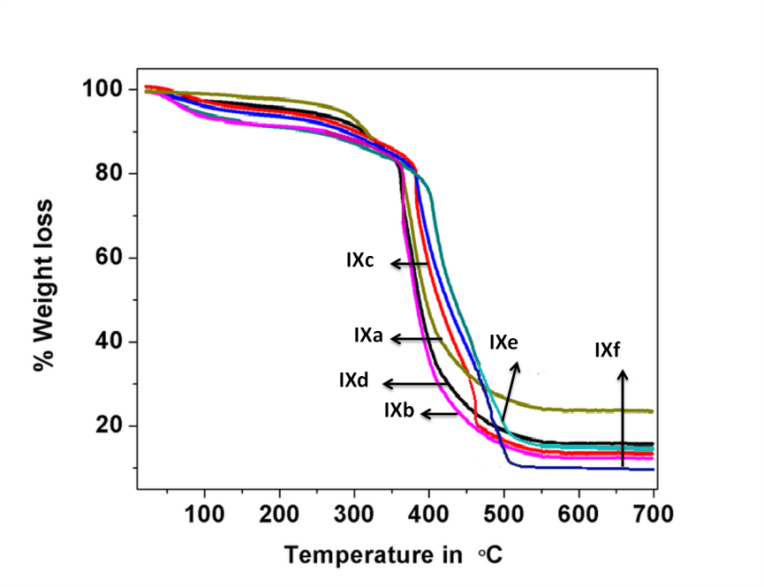
*

**Figure S12.**TGA analytical curve for **(IXa-f).**
